# Ultra-High Molecular Weight Polyethylene Suture and Tape Show Similar Biomechanical Properties in Inside-Out Repair of Lateral Meniscus Tears in a Porcine Model

**DOI:** 10.1016/j.asmr.2024.101071

**Published:** 2024-12-16

**Authors:** José Leonardo Rocha de Faria, Igor Farias de Araújo, Arthur Paiva Grimaldi Santos, Mariana Radulski, Douglas Mello Pavão, Ari Digiacomo Ocampo More, Alan de Paula Mozella, Geraldo da Rocha Motta Filho, João Antonio Matheus Guimarães, Rodrigo Salim, Carlos Rodrigo de Mello Roesler

**Affiliations:** a Knee Surgery Center, National Institute of Traumatology and Orthopedics (INTO), Rio de Janeiro, RJ – Brazil; b School of Medicine from University of São Paulo – USP Ribeirão Preto, SP – Brazil; c Research Division - National Institute of Traumatology and Orthopedics (INTO), Rio de Janeiro, RJ – Brazil; d Biomechanical Engineering Laboratory of Federal University of Santa Catarina, SC – Brazil; e Medical School of Federal University of Santa Catarina, SC – Brazil

## Abstract

**Purpose:**

To compare the stiffness, laxity, and failure rate of ultra-high molecular weight polyethylene (UHMWPE) suture to suture tape measuring 0.15 × 1 mm used in 2 vertical inside-out suture points for the treatment of longitudinal meniscal lesions.

**Methods:**

Twenty-eight porcine knees were selected for this controlled biomechanical evaluation in a laboratory setting. The knees were divided into 2 groups: group S (vertical inside-out meniscal sutures using UHMWPE suture) and group T (vertical inside-out meniscal sutures using UHMWPE tape). A 1.5-cm longitudinal lesion was created in the lateral meniscus, followed by 2 vertical sutures on the femoral surface of the meniscus. Biomechanical analyses were performed using a testing machine, including cyclic loading tests and load-to-failure tests.

**Results:**

Stiffness at the 5th cycle was 21.1 ± 1.8 N/mm for group T and 19.2 ± 2.0 N/mm for group S (*P* = .013). At the 30th cycle, stiffness was 26.1 ± 2.5 N/mm for group T and 23.1 ± 2.4 N/mm for group S (*P* = .003). At ultimate load to failure, stiffness was 26.5 ± 1.6 N/mm for group CS and 25.0 ± 1.6 N/mm for group T (*P* = .918). The results showed no significant difference in lesion widening after cyclic testing or in load-to-failure between the 2 groups.

**Conclusions:**

Both the meniscal suture tape and the ultra-high strength suture exhibit similar biomechanical behaviors under radial loads, making both viable options for meniscal repair. The suture tape demonstrated significantly greater stiffness during the test cycles, which can offer more stable initial fixation. However, the actual differences were small, which limits the clinical relevance of the findings.

**Clinical Relevance:**

The findings of this study provide evidence that, compared with UHMWPE suture, suture tape may offer superior initial stability, which is crucial for the healing process of meniscal tissue.

Meniscal injuries are among the leading types of injuries that can occur in the knee joint. Epidemiologic studies estimate their incidence at approximately 30% of all acute injuries affecting this joint.^1^ The primary functions of this joint tissue are shock absorption, load transmission, joint stability, and maintenance of joint homeostasis, participating in the lubrication and nutrition of articular cartilage.^2^, ^3^, ^4^, ^5^, ^6^

Most injuries to this type of tissue require surgical treatment.^7^ Current knowledge points to the superiority of meniscal repair compared with meniscectomy. Resected meniscal tissue is significantly correlated with an increase in broad and severe chondral lesions, according to a study that evaluated this type of surgery in American football players.^8^ Therefore, meniscal repair should always be attempted, if possible.

The lateral meniscus plays a fundamental role in stability and load distribution in the knee.^1^ Injuries to this structure can lead to painful symptoms, decreased joint function, and potentially the development of osteoarthritis.^1^^,^^3^^,^^4^ Meniscal repair is an essential procedure to restore the integrity and stability of the knee joint.

Traditionally, the therapeutic approach for meniscus injuries involved partial or total resection of the damaged tissue. However, recent evidence has highlighted the importance of long-term meniscal preservation. In this context, repair techniques have become more favored.^4^, ^5^, ^6^ In recent decades, there has been growing interest in less-invasive and more effective techniques for treating meniscal lesions.^1^, ^2^, ^3^

Another point to consider when evaluating meniscal repair is the type of suture material to be used in surgical treatment. Tapes or sutures can be used, which can be multifilament or monofilament, absorbable or nonabsorbable. On the basis of the results of previous studies, such as those by Feucht et al.,^9^ Matthews et al.,^10^ and Bhatia et al.,^11^ it is estimated that tapes may possess more resistant and rigid properties than ultra-high molecular weight polyethylene (UHMWPE) sutures. However, few studies address this topic, and little is known about which suture material behaves biomechanically better when performing traditional vertical inside-out meniscal sutures.

The purpose of this study was to compare the stiffness, laxity, and failure rate of UHMWPE suture to suture tape measuring 0.15 × 1 mm used in 2 vertical inside-out suture points for the treatment of longitudinal meniscal lesions. We hypothesized that the tape suture would demonstrate superior biomechanical properties compared with the suture. This study was conducted in a controlled biomechanical evaluation laboratory. Statistical and biomechanical analyses were performed at a biomechanical engineering laboratory.

## Methods

Authorization from the animal ethics committee was not required, as the evaluation was on porcine knees acquired from commercial food establishments. Twenty-eight porcine knees were selected from hybrid animals, approximately 6 months old and weighing approximately 105 kg. The knees were kept at room temperature until dissection. The femur was carefully resected by one of the authors of this work, avoiding damage to the lateral meniscus during the dissection. Any knee that exhibited injury to the meniscus as a result of dissection or from previous injuries was excluded from the analysis.

### Lesion Preparation and Meniscal Suture Technique

**Fig 1:**
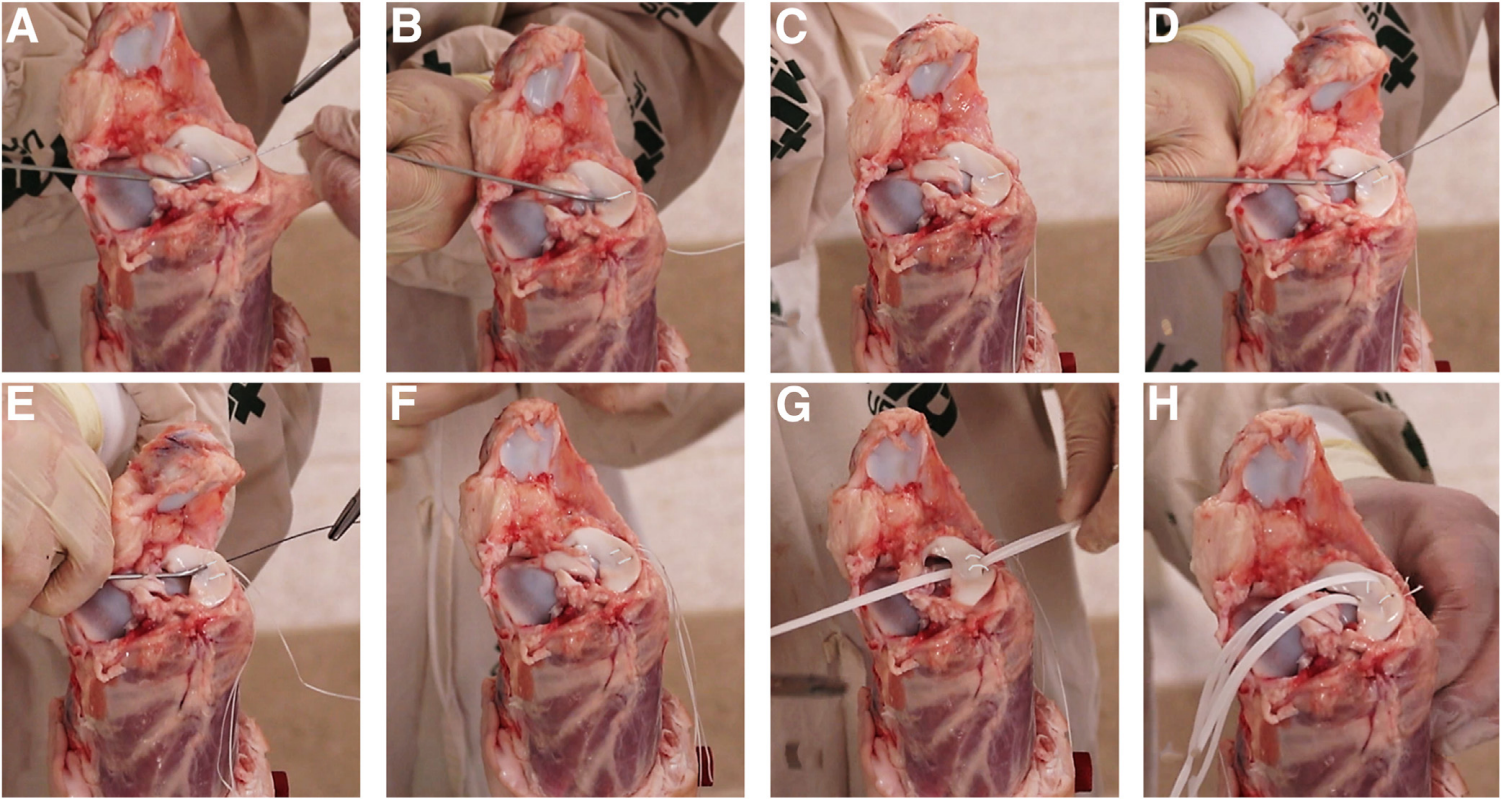
Right porcine knee. Step-by-step process of meniscal repair using 2 parallel sutures with the inside-out technique, using the Protector Meniscus device (Arthrex). (A) The first step involves positioning the Protector Meniscus cannula near the proximal edge of the longitudinal tear on the body of the lateral meniscus. With the cannula in place, we insert the needle into the proximal edge of the tear. (B) The cannula is then positioned on the distal edge of the tear. (C) The first vertical suture is placed without tying the knots. (D) The second parallel suture is initiated by positioning the cannula on the proximal edge of the tear, followed by needle insertion. (E) The cannula is positioned again on the distal edge of the tear. (F) Both vertical sutures are placed without knots. (G) Two traction tapes are passed through the tear. (H) Finally, the sutures are tied.

**Fig 2:**
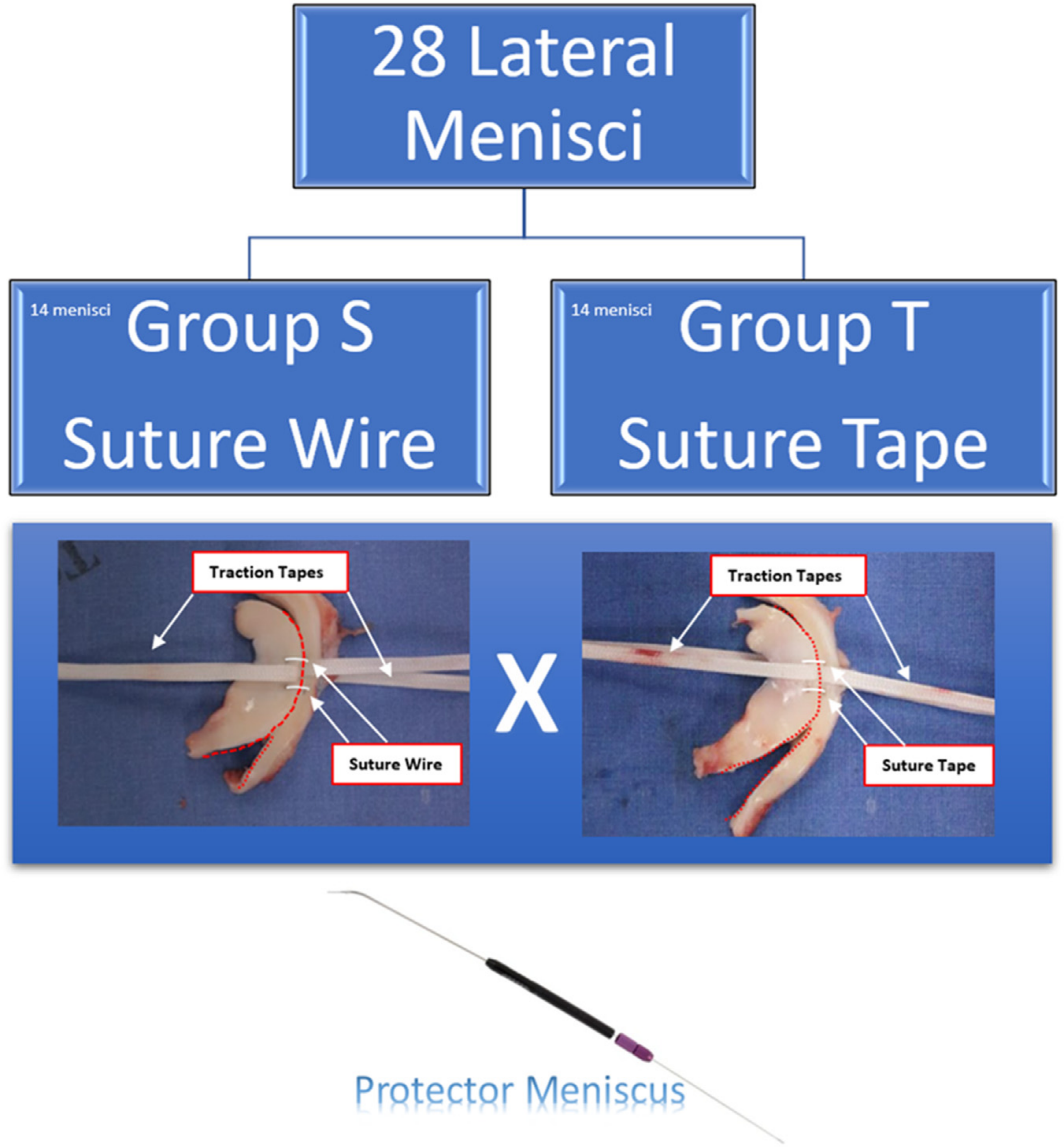
Schematic illustration of the 2 study groups. S group, with 14 medial menisci, submitted to 2 vertical inside-out meniscal repair with suture, and T group, with 14 medial menisci, submitted to 2 vertical inside-out meniscal repair with Suture Tape. The both groups used the Protector Meniscus device (Arthrex).

**Fig 3:**
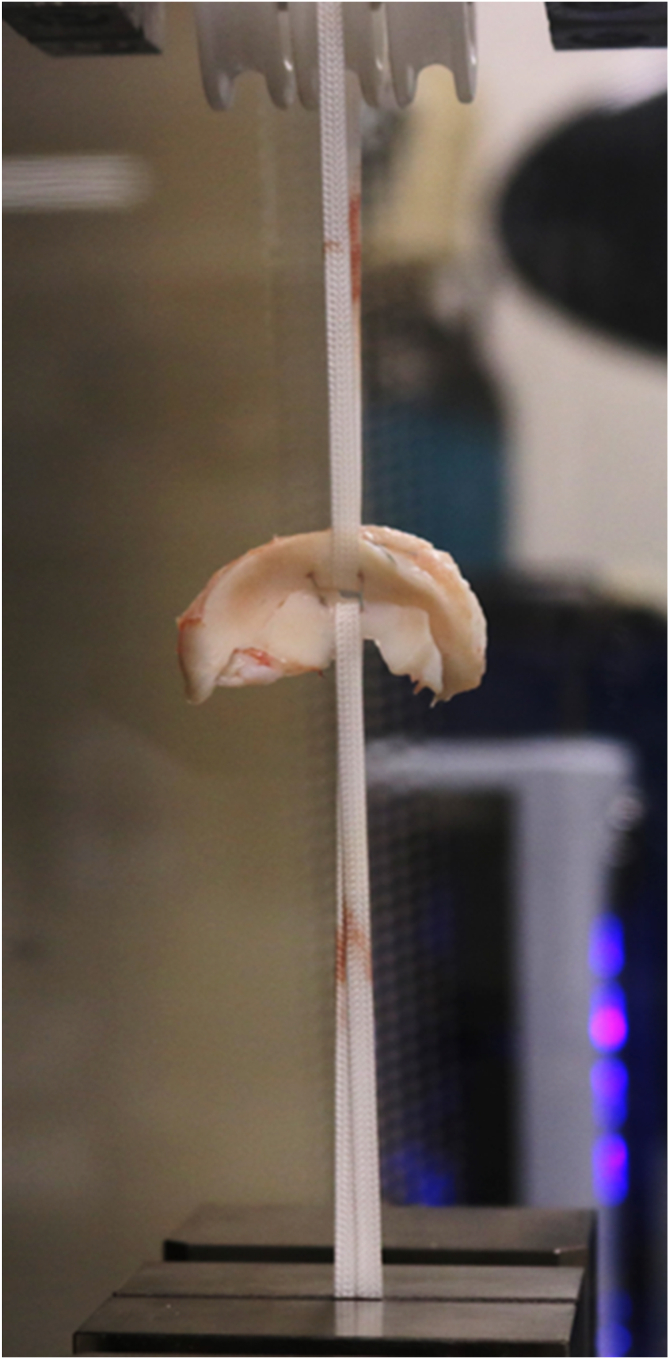
Lateral menisci of an right porcine knee, were prepared for biomechanical analysis on a universal testing machine.

The knees were divided into 2 groups: group S with traditional vertical inside-out meniscal suture using UHPMWE suture 2-0 (S-Fiber, Síntegra Surgical Sciences, Pompéia, SP, Brazil), and group T with traditional vertical inside-out meniscal suture using UHPMWE mini suture tape (0.08 × 0.45 mm) (S-Tape, Síntegra Surgical Sciences, Pompéia, SP, Brazil). A longitudinal lesion of 1.5 cm in length was made 4 mm from the boundary between the meniscus and the capsule at the location of the lateral meniscus body. Two vertical points were made on the femoral side of the meniscus (Fig 1 A-H), each equidistant from each other by about 3 to 4 mm. The Protector Meniscus suture device (Arthrex, Naples, FL) was used for meniscal suturing in both groups (Figs 1 and 2). After passing the 2 sutures, the stitches were tensioned individually; once the first was secured, the second was tensioned. We performed 5 square knots, using 2 half hitches, a locking half hitch, and then 2 additional alternating locking half hitches, as described by Matthews et al. 2020^10^ and Rocha de Faria et al. 2023.^12^ The same researcher (J.L.R.F.) performed all the stitches in all groups, consistently applying the same tension and following the aforementioned sequence to minimize variability in knot security. Two traction tapes (S-tape thickness 0.70 mm, and width 1.10 mm, made of UHMWPE [Teleflex]) were passed between the sutures, and these tapes were positioned in the biomechanical apparatus to radially pull the meniscal edges in opposite directions in an attempt to cause suture failure. After suturing was completed, the lesion was extended completely to the meniscal extremities so that only the suture sutures maintained contact between the injured meniscal edges (Fig 3). This ensured that the biomechanical tests were not influenced by any type of tissue maintaining contact between the meniscal injuries.

The traction tapes were fixed to the standard device to maintain the tapes with opposite radial tensions (Figs 2 and 3). The traction device was attached to a load cell fixed to a universal testing machine (ZwickRoell Z2.5TN, Ulm, Germany). The specimens were kept in physiological solution until testing (Fig 3).

The displacement was recorded using the machine's integrated displacement measurement system, which has a maximum uncertainty of ±0.28%. System conformity was assessed without the influence of the meniscus, enabling more accurate post-test data processing to ensure that the displacement measurements closely reflect actual conditions. The load cell used in the experiment has a maximum capacity of 2.5 kN, with a measurement accuracy of ±0.11%.

Before cyclic loading, a tension preload of 5 N was applied for 30 seconds, followed by 30 loading cycles ranging from 5 to 30 N, with a triangular waveform at 0.25 Hz in displacement control. This cyclic protocol has been used by other investigators^10^^,^^13^, ^14^, ^15^, ^16^ and previous studies showed stabilization of the displacement-versus-time curve between 20 and 30 cycles.^10^^,^^13^^,^^14^^,^^16^ The choice of the 5th cycle allows us to evaluate the construct's initial behavior, capturing any potential early deformation or set-in. In contrast, the 30th cycle reflects the stabilized phase, which is crucial for understanding the long-term performance of the repair. Reporting these 2 points enables us to capture both the early and stabilized responses of the construct under cyclic loading. Upon completion of cyclic loading, each specimen underwent a load-to-failure test at 5 mm/s. During this test, construct stiffness was determined from the linear region of the load versus displacement curve endpoints. The failure mode was visually identified by inspecting the samples at the end of each test. The first suture to fail defined the specific failure mode.

Data distribution probability was considered normal for both groups on the basis of previous studies. Group variance was analyzed using the F-test, and it was found that the groups did not differ significantly. Thus, mean differences were compared using the Student *t* test, assuming equal variance. Failure modes were assessed using the Fisher exact test. Three tests were performed with 2 variables each to determine whether a significant relationship existed between the categorical variables. All tests were performed using statistical analysis software (RStudio, version 1.1.456). Statistical significance was set at *P* < .05.

The main statistical analysis aimed to test the null hypothesis (H0) that the true mean differences (μ) in various biomechanical aspects for group S and group T are equal to zero. The alternative hypothesis (H1) is that the true mean differences are not equal to zero. The analyses addressed the gap widening in the meniscus, the ultimate failure load, and the stiffness at the 5th and 30th cycles, as well as the stiffness at the load leading to failure.

## Results

**Fig 4:**
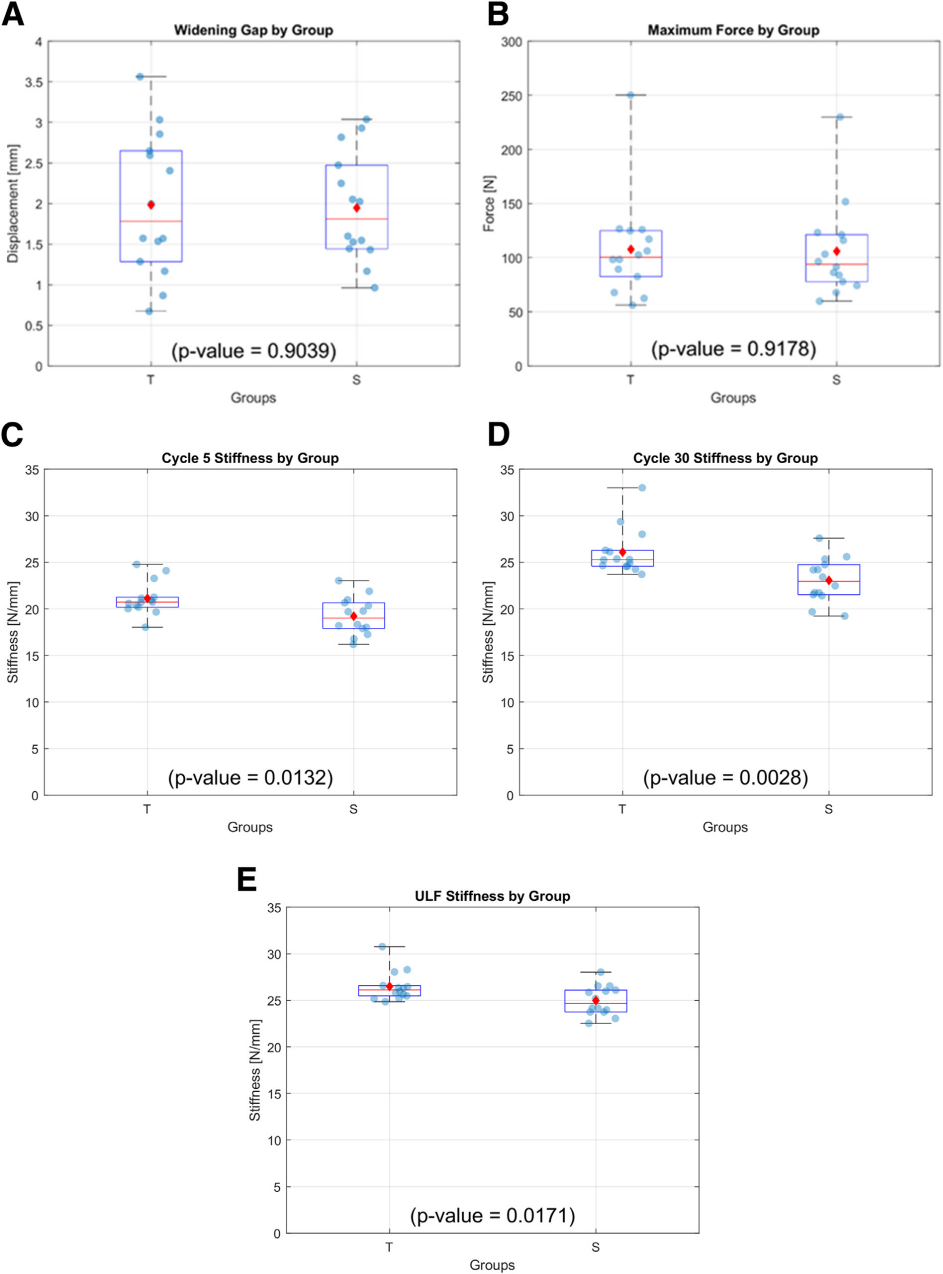
(A) Boxplot showing data for widening at the meniscus repaired site after 30 cycles of preconditioning loading. (B) Ultimate failure load at the load-to-failure test phase. (C-E) System stiffness at the 5th cycle at the 30th cycle and at the ultimate load leading to failure. On the box, the central horizontal line indicates the median, and the bottom and top edges of the box indicate the 25th and 75th percentiles, respectively. The whiskers extend to the most extreme data points. The diamond-shaped dot represents the mean value, and the spherical dots represent each data point.

The mean and standard deviation of the lesion widening after cyclic testing for group T was 1.98 ± 0.88 mm and 1.95 ± 0.67 for group S. Student *t*-test results showed no significant difference in means between both groups (*P* = .904). In the load-to-failure test, the ultimate failure load was 107.7 ± 47.1 N and 105.9 ± 43.6 N in groups T and S, respectively. There was no significant difference between the 2 groups (*P* = .918). The stiffness of the system at the 5th cycle was 21.1 ± 1.8 N/mm for group T and 19.2 ± 2.0 N/mm for group S with significant statistical difference between groups (*P* = .013). The stiffness of the system at the 30th cycle, the last of the preconditioning cycles, was 26.1 ± 2.5 N/mm for group T and 23.1 ± 2.4 N/mm for group S. Significant statistical difference between groups was detected (*P* = .003). The stiffness of the system at the ultimate load leading to failure for group CS was 26.5 ± 1.6 N/mm and for group T was 25.0 ± 1.6 N/mm, there was no significant statistical difference between groups (*P* = .017). However, it should be noted that there is considerable overlap in the box and whisker plot for both groups, suggesting that although statistical significance was observed for the initial stiffness, the actual effect size may be small. The results are illustrated in Figure 4 and outlined in Table 1. Table 2 and Table 3 present the observed frequencies for failure modes, and p-value for fisher exact test comparison between groups and failure modes.

**Table 1:** Mean Widening Gap After 30 Cycles of Cyclic Loading, the Mean Ultimate Failure Load During Load-to-Failure Test, and System Stiffness at Various Phases of the Biomechanical Test

	Widening Gap After 30 Cycles, mm	Ultimate Failure Load, N	Stiffness at Cycle 5, N/mm	Stiffness at Cycle 30, N/mm	Ultimate Load Stiffness, N/mm
**T (n = 14)**	1.98 ± 0.88	107.7 ± 47.1	21.1 ± 1.8	26.1 ± 2.5	26.5 ± 1.6
**S (n = 14)**	1.95 ± 0.67	105.9 ± 43.6	19.2 ± 2.0	23.1 ± 2.4	25.0 ± 1.6
***P* value**	.904	.918	.013	.003	.017

NOTE. Values are given with ± standard deviation. In the last row, *P* value for the *t*-test comparison is shown.

**Table 2:** Observed Frequencies for Failure Modes

	Suture Breakage	Suture Pull-out	Knot Failure
**T (n = 14)**	4	1	9
**S (n = 14)**	0	1	13

**Table 3:** The *P* Value for Fisher Exact Test Comparison

Suture Breakage × Suture Pull-out	Suture Pull-out × Knot Failure	Suture Pull-out × Knot Failure
0.333	0.096	1

NOTE. There were no statistically significant differences between groups with all *P* values greater than 0.05.

## Discussion

The findings of this study indicate that both the meniscal suture tape and the ultra-high-strength suture exhibit similar biomechanical behaviors under radial loads, as evidenced by the lack of differences in lesion widening after cyclic testing and load-to-failure. However, a difference in system stiffness was observed during the test cycles, with the suture tape demonstrating greater stiffness compared with the suture at both the 5th and 30th cycles. It is important to note that greater stiffness does not necessarily equate to better outcomes. Although the increased stiffness of the suture tape may contribute to more stable initial fixation, which could potentially benefit the meniscal healing process, this does not imply that greater stiffness is always preferable. Further research is required to determine the clinical significance of this stiffness difference, as optimal (not maximal) stiffness is often the goal in orthopedic repair, such as in fracture fixation osteosynthesis.

The study by Iuchi et al.^17^ contributes to the understanding of the biomechanics of meniscal repair, as it analyzed different suturing techniques, methods, and the number of sutures, highlighting the superiority of the inside-out technique in terms of fixation stability compared with the all-inside technique and the importance of the vertical suture. Our study focuses on the comparison between ultra-resistant suture thread and thinner tape, demonstrating similar results between the tested materials. Both studies offer valuable information to guide the choice of techniques and materials for meniscal repair, providing complementary insights into the biomechanical aspects involved.

In a similar study, Yokoi et al.^18^ found analogous results when comparing 3 different types of material for meniscal suturing. The authors found that the tape group, with its thinner profile, may suggest superior clinical outcomes compared to traditional suture threads.

Another theoretical advantage of using tapes is the potential for less cartilage abrasion since their thinner profile may generate less damage compared to thicker suture threads. Venjakob et al.^19^ conducted a biomechanical study comparing 3 types of meniscal suture threads, and they found better outcomes with monofilament polydioxanone thread, especially when the suture configuration matched the movement of the meniscal plug. It is believed that if a similar study were conducted with meniscal suture tapes, they would likely show better results when compared with multifilament high-resistance suture threads. Finally, in the clinical setting, it is rare for the suture itself to be the reason for failure. Tissue quality, tear configuration, repair technique, and biology are important factors that were not addressed in this study.

In the biomechanical comparison by Matthews et al.^10^ of 9 different meniscal suture materials, including tapes and high-resistance threads, the study also noted a trend toward greater stiffness and higher maximum load for failure in tapes. This suggests a potential biomechanical advantage for the use of tapes in certain settings.^10^

Clinical retrospective studies, such as those by Hiranaka et al.^20^ and Furumatsu et al.,^21^ support our biomechanical findings, showing no significant difference between the types of suture materials and the techniques of meniscal repair when evaluating postoperative clinical outcomes and meniscal healing.

### Limitations

As limitations of our study, we cite the use of porcine models, which, while similar, are not identical to human meniscal tissue. In addition, the radial traction biomechanical assessment does not replicate the exact forces meniscal tissue is subjected to postoperatively.

## Conclusions

We conclude that both the meniscal suture tape and the ultra-high-strength suture exhibit similar biomechanical behaviors under radial loads, making both viable options for meniscal repair. However, the suture tape demonstrated significantly greater stiffness during the test cycles, which can offer more stable initial fixation. However, the actual differences were small, which limits the clinical relevance of the findings.

## Disclosures

The authors declare the following financial interests/personal relationships which may be considered as potential competing interests: J.L.R.d.F. reports equipment, drugs, or supplies provided by Sintegra Surgical Sciences; consulting or advisory, speaking and lecture fees, and travel reimbursement with Sintegra Surgical Sciences; and Junior Editor of the *Revista Brasileira de Ortopedia*. G.d.R.M.F. is editor in chief of the *Revista Brasileira de Ortopedia*. J.A.M.G. and A.d.P.M. are members of Editorial board of the *Revista Brasileira de Ortopedia*. All other authors (I.F.d.A., A.P.G.S., M.R., D.M.P., A.D.O.M., R.S., C.R.d.M.R..) declare that they have no known competing financial interests or personal relationships that could have appeared to influence the work reported in this paper.

## References

[1] Majewski M., Susanne H., Klaus S. (2006). Epidemiology of athletic knee injuries: A 10-year study. Knee.

[2] Cameron H.U., Macnab I. (1972). The structure of the meniscus of the human knee joint. Clin Orthop.

[3] Makris E.A., Hadidi P., Athanasiou K.A. (2011). The knee meniscus: Structure–function, pathophysiology, current repair techniques, and prospects for regeneration. Biomaterials.

[4] Newman A.P., Anderson D.R., Daniels A.U. (1989). Mechanics of the healed meniscus in a canine model. Am J Sports Med.

[5] Tissakht M., Ahmed A.M., Chan K.C. (1996). Calculated stress-shielding in the distal femur after total knee replacement corresponds to the reported location of bone loss. J Orthop Res.

[6] Zhu W., Chern K.Y., Mow V.C. (1994). Anisotropic viscoelastic shear properties of bovine meniscus. Clin Orthop.

[7] Bansal S., Floyd E.R., Kowalski M. (2021). Meniscal repair: The current state and recent advances in augmentation. J Orthop Res.

[8] Chahla J., Cinque M.E., Godin J.A. (2018). Meniscectomy and resultant articular cartilage lesions of the knee among prospective National Football League Players: An imaging and performance analysis. Am J Sports Med.

[9] Feucht M.J., Grande E., Brunhuber J. (2013). Biomechanical evaluation of different suture techniques for arthroscopic transtibial pull-out repair of posterior medial meniscus root tears. Am J Sports Med.

[10] Matthews J.R., Wang J., Zhao J. (2020). The influence of suture materials on the biomechanical behavior of suture-meniscal specimens: A comparative study in a porcine model. Knee Surg Relat Res.

[11] Bhatia S., Civitarese D.M., Turnbull T.L. (2016). A novel repair method for radial tears of the medial meniscus: Biomechanical comparison of transtibial 2-tunnel and double horizontal mattress suture techniques under cyclic loading. Am J Sports Med.

[12] Rocha de Faria J.L., Santos A.P.G., Pavão D.M. (2023). Continuous vertical inside-out versus traditional vertical inside-out meniscal repair: A biomechanical comparison. Orthop J Sports Med.

[13] Bisson L.J., Manohar L.M. (2010). A biomechanical comparison of the pullout strength of No. 2 FiberWire suture and 2-mm FiberWire tape in bovine rotator cuff tendons. Arthroscopy.

[14] Bisson L.J., Manohar L.M., Wilkins R.D. (2008). Influence of suture material on the biomechanical behavior of suture-tendon specimens: A controlled study in bovine rotator cuff. Am J Sports Med.

[15] Ma C.B., Comerford L., Wilson J. (2006). Biomechanical evaluation of arthroscopic rotator cuff repairs: Double-row compared with single-row fixation. J Bone Joint Surg Am.

[16] MA C.B., MacGillivray J.D., Clabeaux J. (2004). Biomechanical evaluation of arthroscopic rotator cuff stitches. J Bone Jt Surg.

[17] Iuchi R., Mae T., Shino K., Matsuo T. (2017). Biomechanical testing of transcapsular meniscal repair. J Exp Orthop.

[18] Yokoi H., Mae T., Iuchi R. (2017). Novel flat and wide meniscal repair material improves the ultimate load of knot breakage in a porcine trans-capsular meniscal repair model. J Exp Orthop.

[19] Venjakob A.J., Föhr P., Henke F. (2019). Influence of sutures on cartilage integrity: Do meniscus sutures harm cartilage? An experimental animal study. Arthroscopy.

[20] Hiranaka T., Furumatsu T., Kamatsuki Y. (2020). Early chondral damage following meniscus repairs with anterior cruciate ligament reconstruction. Asia-Pac J Sports Med Arthrosc Rehabil Technol.

[21] Furumatsu T., Okazaki Y., Kodama Y. (2019). Pullout repair using modified Mason-Allen suture induces better meniscal healing and superior clinical outcomes: A comparison between two surgical methods. Knee.

